# A five-gene reverse transcription-PCR assay for pre-operative classification of breast fibroepithelial lesions

**DOI:** 10.1186/s13058-016-0692-6

**Published:** 2016-03-09

**Authors:** Wai Jin Tan, Igor Cima, Yukti Choudhury, Xiaona Wei, Jeffrey Chun Tatt Lim, Aye Aye Thike, Min-Han Tan, Puay Hoon Tan

**Affiliations:** Division of Biodevices and Diagnostics, Institute of Bioengineering and Nanotechnology, 31 Biopolis Way, The Nanos, #04-01, Singapore, 138669 Republic of Singapore; Department of Pathology, Singapore General Hospital, 20 College Road, Academia, Level 7, Diagnostics Tower, Singapore, 169856 Republic of Singapore; Duke-NUS Graduate Medical School, 8 College Road, Singapore, 169857 Republic of Singapore

**Keywords:** Phyllodes tumors, Fibroadenomas, Fibroepithelial lesions, Prediction, Multigene assay, Core biopsy, FFPE

## Abstract

**Background:**

Breast fibroepithelial lesions are biphasic tumors and include fibroadenomas and phyllodes tumors. Preoperative distinction between fibroadenomas and phyllodes tumors is pivotal to clinical management. Fibroadenomas are clinically benign while phyllodes tumors are more unpredictable in biological behavior, with potential for recurrence. Differentiating the tumors may be challenging when they have overlapping clinical and histological features especially on core biopsies. Current molecular and immunohistochemical techniques have a limited role in the diagnosis of breast fibroepithelial lesions. We aimed to develop a practical molecular test to aid in distinguishing fibroadenomas from phyllodes tumors in the pre-operative setting.

**Methods:**

We profiled the transcriptome of a training set of 48 formalin-fixed, paraffin-embedded fibroadenomas and phyllodes tumors and further designed 43 quantitative polymerase chain reaction (qPCR) assays to verify differentially expressed genes. Using machine learning to build predictive regression models, we selected a five-gene transcript set (*ABCA8*, *APOD*, *CCL19*, *FN1*, and *PRAME*) to discriminate between fibroadenomas and phyllodes tumors. We validated our assay in an independent cohort of 230 core biopsies obtained pre-operatively.

**Results:**

Overall, the assay accurately classified 92.6 % of the samples (AUC = 0.948, 95 % CI 0.913–0.983, *p* = 2.51E-19), with a sensitivity of 82.9 % and specificity of 94.7 %.

**Conclusions:**

We provide a robust assay for classifying breast fibroepithelial lesions into fibroadenomas and phyllodes tumors, which could be a valuable tool in assisting pathologists in differential diagnosis of breast fibroepithelial lesions.

**Electronic supplementary material:**

The online version of this article (doi:10.1186/s13058-016-0692-6) contains supplementary material, which is available to authorized users.

## Background

Fibroadenomas and phyllodes tumors are fibroepithelial lesions of the breast, characterized by proliferation of both epithelial and stromal components. Fibroadenomas are more commonly encountered on core biopsies than the rarer phyllodes tumors (approximately 20 % and <1 % of breast core needle biopsies respectively) [1, 2]. The preoperative distinction between the two lesions has significant impact on subsequent treatment. The current recommended management for phyllodes tumor diagnosed on core biopsy is wide excision without axillary staging regardless of grade [3]. Conversely, fibroadenomas are observed conservatively or, if tumors are larger than 2 cm, may be simply excised without achieving negative surgical margins [3]. This approach is due to the indolent behavior of fibroadenomas, despite sporadic reports of recurrences [4, 5], while phyllodes tumors have unpredictable outcomes with malignant tumors potentially progressing to metastasis and mortality [6–10]. It has been challenging separating cellular fibroadenoma from benign phyllodes tumor due to overlapping histological features, and this is particularly problematic on limited material of core biopsies, which may lead to over- or under-treatment for some patients, resulting in unnecessary anxiety and cost.

Several studies have proposed differentiating histological features such as stromal cellularity, stromal overgrowth, fragmentation, subepithelial condensation and presence of adipose tissue within stroma on core biopsies being indicative of phyllodes tumor [11–13].However, interpretation of these parameters is subjective, with interobserver variation and only moderate reproducibility between pathologists [11, 14]. Varied reports of immunohistochemical markers used in distinguishing phyllodes tumors from fibroadenomas suggest a lack of consensus and objectivity in assessing the expression of these biomarkers. Some authors reported Ki-67 expression to be helpful in diagnosing phyllodes tumors [15–17] but there are reports to the contrary [18, 19]. Lin et al*.* suggested a combination immunoscore of p16-INK4a and retinoblastoma-associated protein (pRB) [20] while Maity et al*.* reported expression of collagen I, III and CD105-positive microvessel density as parameters to differentiate the two lesions [21]. The vast majority of these studies were not conducted using pre-operative biopsies, which is where key management decision is required.

We set out to identify a useful molecular signature to help differentiate fibroadenomas from phyllodes tumors using pre-operative core biopsies to improve prediction of the final diagnosis.

## Methods

### Training set for assay development

The study received approval from the Centralized Institutional Review Board (CIRB 2005/002/F). As this was a retrospective study with anonymized cases, no specific patient consent was individually required. Forty-eight samples (24 fibroadenomas and 24 phyllodes tumors) were first employed as the training set for assay development. These included 10 paired core biopsies and surgical samples (20 samples), and 28 independent core and excisional samples from 38 patients (Table 1 and Additional file 1: Table S1). These formalin-fixed, paraffin-embedded (FFPE) samples were randomly selected from cases diagnosed at the Department of Pathology, Singapore General Hospital from 2008 to 2012. Hematoxylin and eosin (H&E)-stained slides were retrieved and reviewed. Phyllodes tumor was defined when there were well-developed fronds accompanied by increased stromal cellularity as opposed to fibroadenomas in which epithelial and stromal components were arranged in either intracanalicular or pericanalicular patterns without fronds or stromal hypercellularity. Differences in clinical features between fibroadenomas and phyllodes tumors were assessed with Mann–Whitney *U* test and Fisher’s exact test.

**Table 1:** Clinical features of the training cohort from 38 patients

Features	Fibroadenomas (n = 19)	Phyllodes tumors (n = 19)	*p* value
Age			
Median (range)	35 (17–80)	44 (18–64)	0.09
Size			
Median (range)	25 (15–50)	65 (25–220)	< 0.001
Ethnicity, n (%)			0.2
Chinese	13 (68.4)	11 (57.9)	
Malay	0 (0.0)	4 (21.0)	
Indian	2 (10.5)	1 (5.3)	
Others	4 (21.1)	3 (15.8)	
Histology			
Simple fibroadenoma	15^a^		
Complex fibroadenoma	4^c^		
Benign phyllodes tumor		13^b^	
Borderline phyllodes tumor		3^c^	
Malignant phyllodes tumor		3^c^	

^a^Four paired core biopsies and surgical excisions

^b^Three paired core biopsies and surgical excisions

^c^One paired core biopsy and surgical excision

### Expression profiling by Whole-Genome DASL® High Throughput (HT) Assay

Representative tumor areas were identified of which three to seven sections of 10-μm-thick sections from the same FFPE tumor block were obtained, deparaffinized and macrodissected. RNA was extracted using the RNeasy FFPE kit (Qiagen, Hilden, Germany) and quantified by Nanodrop Spectrophotometer (Thermo Fisher Scientific, Waltham, MA, USA). A total of 100 ng was used for quality assessment by real-time amplification of the *RPL13A* gene (forward primer, 5’-CACTTGGGGACAGCATGAG-3’, and reverse primer, 5’-GTAACCCCTTGGTTGTGCAT-3’) using the Power SYBR® Green RNA-to-CT™ 1-Step Kit (Life Technologies, Carlsbad, CA, USA) on a CFX96™ Real-Time PCR instrument (Bio-Rad Laboratories, Hercules, CA, USA). Samples with threshold cycle (Ct) below 29 were further subjected to quality assessment on a bioanalyzer. Eligible samples were submitted for expression profiling on the Whole-Genome DASL® HT Assay (Illumina, Inc., San Diego, CA, USA) at the Biopolis Shared Facilities A*Star, Singapore. The assay interrogates 29,377 features using the HumanHT-12 v4 BeadChip (Illumina, Inc.). Quantile-normalized gene expression data pre-analyzed using GenomeStudio® (Illumina, Inc.) was delivered. Data are available through GEO [GEO: GSE78071].

### Selection of normalization genes and differentiating genes

Normalization genes were selected based on the smallest value of coefficient of variation among all samples. Differentiating genes were selected using the Significance Analysis of Microarrays package [22] and filtered based on the following criteria: (1) q-value less than 0.05; (2) mean difference of expression above 500; (3) R-fold above 1.5 (for genes highly expressed in phyllodes tumors) or less than 0.67 (for genes highly expressed in fibroadenomas).

### Design of quantitative polymerase chain reaction (qPCR) assay

cDNA was synthesized from 1 μg of RNA using the High-Capacity cDNA Reverse Transcription Kit (Applied Biosystems®, Life Technologies, Carlsbad, CA, USA). Each qPCR assay consisted of 1X Power SYBR® Green PCR Master Mix (Life Technologies), 0.5 μM of forward and reverse primer each and 1 μl of 10-fold diluted cDNA as a template in a final total volume of 10 μl. Primers were designed using Primer-BLAST (NCBI, Bethesda, MD, USA) [23] with accession number listed in Additional file 1: Table S2. Non-template control acted as a negative control. Specificity of the amplicons was verified by melt curve analysis.

### Data quantification and model building

Delta Ct (ΔCt) for each gene and sample was quantified as: *ΔCt* = *Ct*(*gene*, *sample*) − *geomean*(*Ct*(*five normalization gene*)). For comparison with expression of the Whole-Genome DASL® HT Assay, ΔCt data was transformed to 2^-ΔCt^ as a positive linear scale and significance of correlation was analyzed with Pearson’s correlation test. Similarly to Cima et al. and Kälin et al*.* [24, 25], we used Random Forest (RF) ensemble classifier [26] to rank the importance of gene transcripts differentiating fibroadenomas and phyllodes tumors on qPCR assays. The top seven performing genes were used to build predictive logistic regression models using exhaustive search for the best model. To this end we used the *glmulti* package [27] with inclusion of the interaction terms. The best model was selected based on the lowest Akaike information criteria (AIC) value [28].

### Validation cohort for model validation

The model of the multigene assay was tested on a separate set of 230 core biopsies with at least 2 years of follow-up. Hematoxylin and eosin (H&E)-stained slides were retrieved and reviewed. The outcome of the multigene assay was compared against the final diagnosis on the corresponding surgical excisions. Cases without subsequent surgical excisions were free from progression for at least 2 years and diagnosis made based on the initial core biopsy was used as the reference instead.

## Results

### Clinical features of training set

**Fig. 1:**
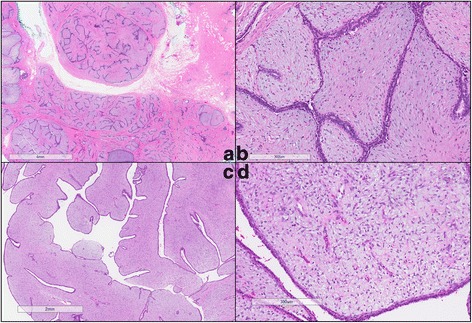
Histology of fibroadenoma and phyllodes tumor on surgical excisions. **a**, **b** An example of a fibroadenoma at low-power (**a**) and high-power (**b**) magnifications. **c**, **d** An example of a benign phyllodes tumor at low-power (**c**) and high-power (**d**) magnifications

The clinical features and histology of the training set are shown in Table 1. Examples of the histological appearances of fibroadenoma and phyllodes tumor are shown in Fig. 1. Phyllodes tumors were significantly larger than fibroadenomas (*p* < 0.001). Median age of patients diagnosed with fibroadenomas and phyllodes tumors was 35 years and 44 years respectively (*p* = 0.09). No significant differences were observed for ethnicity distribution between the two groups of tumors.

### Expression profiling and correlation with qPCR assays

Forty-seven samples (97.9 %) from 37 patients passed the quality control and were profiled successfully. Genes discriminating fibroadenomas and phyllodes tumors are listed in Additional file 1: Table S3. We designed and validated qPCR assays on 43 selected genes. Concordance between expression profiling and the qPCR assays was assessed based on a pilot run on six representative samples (Additional file 1: Table S4). Twenty-three assays with Pearson’s *r* of above 0.6 were further tested on the remaining 40 samples. One case was excluded due to insufficient material after expression profiling.

### Development of a multigene qPCR panel

**Fig. 2:**
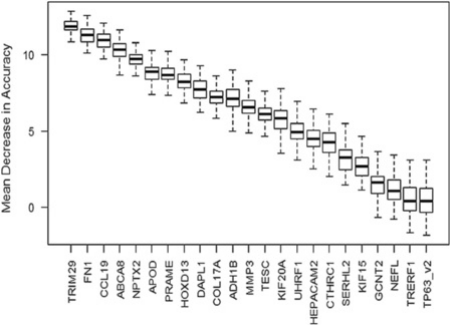
Box plot of mean decrease in accuracy for 100 Random Forest trees generated for each gene. Mean decrease in accuracy measures the importance of each gene to the classification

The results of ΔCt for all 23 qPCR assays were ranked using variable importance feature of the Random Forest classifier (Fig. 2). The seven most important genes in separating fibroadenomas and phyllodes tumors were *TRIM29, FN1, CCL19, ABCA8, NPTX2, APOD* and *PRAME*. A total of 268,435,456 candidate models were identified by *glmulti* based on these seven genes. We employed the genetic algorithm approach in the package to perform automated screening for the best model based on AIC value. A final five-gene model encompassing *APOD*, *ABCA8*, *PRAME*, *FN1*, and *CCL19* with AIC of 14.2 was returned with coefficients as listed in Table 2.

**Table 2:** Coefficients of the best model in predicting diagnosis in the 46-sample set

Genes	Coefficients
*APOD*	2.95575
*APOD:ABCA8*	−0.11934
*PRAME:FN1*	−0.43165
*PRAME:CCL19*	0.08326

### Performance of the model

**Fig. 3:**
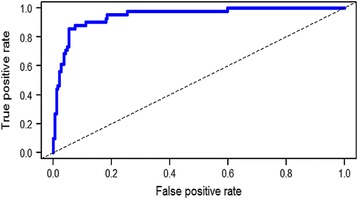
Receiver operating characteristics (ROC) curve of the five-gene model with an area under the curve (AUC) of 0.948 (95 % confidence interval (CI) 0.913–0.983, *p* = 2.51E-19) in predicting diagnosis in the independent set of 230 core biopsies

Patient demographics of the validation cohort of 230 core biopsies are shown in Table 3. Overall the assay was able to predict 213 (92.6 %) cases accurately. The prediction accuracy rates for fibroadenomas and phyllodes tumors were 94.7 % (179/189) and 82.9 % (34/41) respectively (Table 4), with positive (PPV) and negative (NPV) predictive values of 77.3 % and 96.2 %. A receiver operating characteristics curve with an area under the curve (AUC) of 0.948 (95 % confidence interval (CI) 0.913–0.983, *p* = 2.51E-19) was generated, indicating a large effect size in expected diagnostic performance for the five-gene assay (Fig. 3).

**Table 3:** Patient demographics of the validation cohort of 230 core biopsies

Characteristics	*N*	Percentage (%)
Age		
Median	46 years	
Range	15–75 years	
Ethnicity		
Chinese	164	71.3
Malay	23	10.0
Indian	11	4.8
Others	32	13.9
Diagnosis		
Fibroadenoma	189	82.2
with subsequent surgical excisions	58	25.2
without subsequent surgical excisions	131	57
Phyllodes tumors	41	17.8
Benign	22	9.6
Borderline	16	6.9
Malignant	3	1.3

**Table 4:** Performance of the five-gene model in predicting diagnosis in the independent validation cohort of 230 core biopsies

	Diagnosis from pathological report		
Predicted outcome from the five-gene assay	Fibroadenomas	Phyllodes tumors	
Fibroadenomas	179	7	NPV = 0.962
Phyllodes tumors	10	34	PPV = 0.773
	Spec = 0.947	Sen = 0.829	

The five-gene assay has an overall accuracy of 92.6 %, with a sensitivity (sen) of 82.9 % and specificity (spec) of 94.7 %. The positive predictive value (PPV) and negative predictive value (NPV) are 77.3 % and 96.2 % respectively

**Fig. 4:**
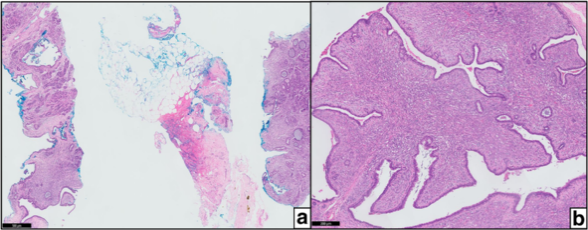
Example of a discordant case containing high epithelium content. The five-gene assay predicted the core biopsies (**a**) as fibroadenoma but the final surgical excision (**b**) was diagnosed as phyllodes tumor on pathological reports

Of the 17 discordant cases (Table 5), seven were diagnosed as phyllodes tumors on pathological reports but were predicted as fibroadenomas on our assay. Upon review of these seven cases, two cases contained high epithelium content (Fig. 4), two were heterogeneous on histology with focal areas suggestive of fibroadenomas, while three other cases were confirmed as phyllodes tumors on review. The other ten of the 17 discordant cases were diagnosed as fibroadenomas on pathological reports but were predicted as phyllodes tumors on our assay. Among these ten cases, six cases had post-operative excisional material available as reference while the remaining four cases were benchmarked against the pre-operative pathological diagnosis. Of the six with excisional material, four were unequivocally fibroadenomas on histology, one was a cellular fibroadenoma without prominent fronds, and one was a fibroadenoma with sclerosing adenosis. Of the four pre-operative biopsies, one was unequivocally fibroadenoma, two cases contained features in keeping with fibroadenoma with hyalinized leafy fronds noted albeit without stromal cellularity, and one was an indeterminate case with focal areas of hemorrhage and high cellularity, which could not be definitively concluded on review.

**Table 5:** Seventeen cases with discordant outcomes between the five-gene assay and pathological diagnosis in the validation cohort. Post-operative diagnoses were used as benchmark reference unless otherwise stated

Sample ID	Five-gene assay	Pathological diagnosis
CB22	FA	Benign PT^a^
CB116	FA	Benign PT
CB95	FA	Benign PT
CB28	FA	Benign PT^a^
CB68	FA	Benign PT^a^
CB120	FA	Borderline PT^a^
CB130	FA	Borderline PT
CB26	PT	Cellular FA
CB77	PT	FA
CB24	PT	FA
CB29	PT	FA
CB82	PT	FA
CB126	PT	FA
CB144	PT	FA^b,c^
CB184	PT	FA^b,c^
CB251	PT	FA^b^
CB258	PT	FA^b,d^

*FA* fibroadenoma, *PT* phyllodes tumor

^a^Pre-operative pathological diagnoses were inconclusive or discordant with post-operative pathological diagnoses (see Table 6 asterisked cases)

^b^Cases of core biopsies without subsequent surgical excisions. Outcome of the five-gene assay was benchmarked against the pre-operative pathological diagnosis

^c^Features in keeping with fibroadenoma with hyalinized leafy fronds noted albeit without stromal cellularity

^d^Focal areas of hemorrhage and high cellularity, diagnosis could not be definitively concluded on review

Among the 230 core biopsies, the pre-operative pathological diagnoses were inconclusive for 22 cases where the term ‘fibroepithelial lesion’ was assigned, and there were three cases where the preoperative diagnoses were incongruous with the post-operative outcome (Table 6). Of these 25 cases, the five-gene assay was 80 % (20/25) accurate in classification with a PPV of 94.7 %.

**Table 6:** Cases with inconclusive pre-operative pathological diagnoses (n = 22), and discordant pre-and post-operative pathological diagnoses (n = 3). Among these cases, the five-gene assay was 80 % (20/25) accurate in predicting the final post-operative outcome

Sample ID	Pathological reports		Outcome from five-gene assay
	Pre-operative	Post-operative	
CB22	FA	Benign PT	FA^a^
CB36	FA	Benign PT	PT
CB111	FA	Benign PT	PT
CB21	FEL	Benign PT	PT
CB31	FEL	Benign PT	PT
CB40	FEL	Benign PT	PT
CB43	FEL	Benign PT	PT
CB57	FEL	Benign PT	PT
CB59	FEL	Benign PT	PT
CB96	FEL	Benign PT	PT
CB99	FEL	Benign PT	PT
CB107	FEL	Benign PT	PT
CB114	FEL	Benign PT	PT
CB20	FEL	Borderline PT	PT
CB42	FEL	Borderline PT	PT
CB55	FEL	Borderline PT	PT
CB73	FEL	Borderline PT	PT
CB78	FEL	Borderline PT	PT
CB101	FEL	Borderline PT	PT
CB115	FEL	Borderline PT	PT
CB122	FEL	Borderline PT	PT
CB28	FEL	Benign PT	FA^a^
CB68	FEL	Benign PT	FA^a^
CB120	FEL	Borderline PT	FA^a^
CB77	FEL	FA	PT^a^

*FA* fibroadenoma, *PT* phyllodes tumor, *FEL* fibroepithelial lesion without definitive assignment into fibroadenoma or phyllodes tumor

^a^Inaccurate classification by the five-gene assay benchmarked against the post-operative pathological diagnosis

## Discussion

Classification of breast fibroepithelial tumors based on differentiating morphological and immunohistochemical features on pre-operative material has been challenging with variable findings across different groups (see Table 7 for summary). Jacobs et al. and Lee et al. first described individual pathological parameters which might help to differentiate fibroadenomas and phyllodes tumors in these limited samples [11, 16]. Jara-Lazaro proposed a combination of histological and immunohistochemical markers to indicate phyllodes tumors on core biopsies [15] but did not weigh the relative importance of each parameter in predicting phyllodes tumors. Morgan addressed this question by proposing a predictive tool including coefficient factors for each parameter to distinguish between fibroadenomas and phyllodes tumors but this has yet to be validated in an independent series of core biopsies [12]. Our study is the first to investigate differentiating features of fibroepithelial lesions on pre-operative material at the molecular level. We have developed a five-gene assay using a systematic approach based on genome-wide expression profiling data and validated the assay in an independent cohort of 230 pre-operative core biopsies of breast fibroepithelial lesions, the largest cohort reported so far. The pre-operative core biopsies were FFPE tissue containing low-quality RNA. Accordingly, our assay has been developed using RNA extracted from limited FFPE materials from core biopsies and thus is expected to perform on such material in the clinical setting.

**Table 7:** Summary of selected studies investigating differential features of fibroadenomas and phyllodes tumors on pre-operative materials

Author	N	Assessment approach	Key findings
Jacobs [16]	16 FAs 12 PTs	Histology Immunohistochemistry	Stromal **cellularity**, **mitoses**, **expression of Ki-67** and **topoisomerase IIα** might help determining phyllodes tumors
Lee [11]	38 FAs 44 PTs	Histology	Features indicating phyllodes tumors:
			(1) increased stromal **cellularity** in at least 50 % of the specimen; (2) stromal **overgrowth**; (3)**fragmentation** and; 4) **presence of adipose** tissue within stroma
Jara-Lazaro [15]	21 FAs 36 PTs	Histology Immunohistochemistry	(1) Marked stromal hypercellularity and nuclear atypia, stromal overgrowth, and ill-defined lesional margins exclusively predicted phyllodes tumor on excision.
			(2) Expression of **Ki67 ≥ 5 %**, **topoisomerase IIα ≥ 5** % and **reduced CD34** correlates significantly with phyllodes tumors.
Morgan [12]	91^a^ FAs 21 PTs	Histology	Proposed two putative predictive tools:
			(1) *Logit P*(*x*) = − 0.9014 (***age***) − 3.61 (***mitosis***) + 11.156
			(2) *Z* = 0.8909(***age***) + 0.0347(% ***stroma***) + 0.5274 (***mitoses***/10*HPF*)
Yasir [13]	37 FAs 27 PTs	Histology	**Stromal mitoses** and/or three or more histological features were helpful in predicting phyllodes tumors on excisions.

*FAs* fibroadenomas, *PTs* phyllodes tumors

^a^Diagnoses not confirmed on excisions

Comparatively in surgical excisional materials, Huang et al. proposed a two-gene test derived from methylation profiling of an 11-gene panel in 86 samples [29], which described an elevated *RASSF1A* and/or *TWIST1* methylation observed in phyllodes tumors as compared to fibroadenomas. They further evaluated the test in a separate validation cohort of 19 samples and reported a sensitivity and specificity of 0.33 and 0.75 respectively, with a PPV and NPV of 0.83 and 0.23. However, development of the test from a pre-selected panel of 11 genes may not be representative and the sample size of the validation cohort was too small to be conclusive. In contrast, our assay has a better sensitivity and specificity at 0.83 and 0.95 despite a lower PPV of 0.77. In a separate study by Kuijper interrogating the transcriptome differences between five fibroadenomas and eight phyllodes tumors, *CTAG1/2*, *PRAME*, *HOXC13*, *ELF5* and *FABP7* were among 96 other transcripts found to be highly differentially expressed between fibroadenomas and phyllodes tumors [30]. More recently, Vidal et al. reported a cluster of 47 epithelial- and luminal-related genes was found to be more expressed in fibroadenomas than phyllodes tumors among 105 breast cancer-related genes studied [31]. Findings from these studies however, were not further deployed as a test to distinguish fibroadenomas from phyllodes tumors on pre-operative materials despite the significant differential expression observed.

The training cohort comprised a mixture of surgical excisions and core biopsies with varying classifications of fibroepithelial lesions, simulating a realistic clinical scenario. Phyllodes tumors comprise benign, borderline and malignant grades on a continuous spectrum [32]. It is important that the assay works across the spectrum although one may argue that the malignant grade of phyllodes tumors is rarely in the histologic differential diagnosis between fibroadenomas and phyllodes tumors, and hence the assay may have little utility in the separation of fibroadenomas from malignant phyllodes tumors. The proportion of malignant phyllodes tumors included in the training cohort concurs with the incidence of malignant phyllodes tumors reported in the literature [33]. Nevertheless, even with the exclusion of malignant phyllodes tumors in the training cohort, differences of expression between fibroadenomas and phyllodes tumors for genes selected for the assay still fall within our selection criteria (R-fold differences above 1.5 and mean differences above 500) and hence would not have altered the assay development outcome. Excluding the three malignant tumor cases in the validation cohort only slightly reduces the sensitivity and PPV from 0.829 and 0.773 to 0.816 and 0.756 respectively. It is not our aim to investigate the differential expression between the phyllodes tumor grades although there is a trend of differences observed between grades in the expression of these five genes (results not shown). The sample sizes of borderline and malignant phyllodes tumors would be too small for meaningful analysis.

Several underlying factors which potentially limit the performance of the assay resulting in 17 discordant outcomes between the assay and pathological diagnosis include tumor heterogeneity and the issue of sampling on core biopsies. These factors may also have contributed to the three discordant pathological diagnoses between pre-operative core and post-operative excision materials. Core biopsies offer insight into only part of a tumor, which may not truly represent its entirety. Also, it is not uncommon for phyllodes tumors to contain areas indistinguishable from fibroadenomas, as seen in two discordant phyllodes tumor cases incorporating focal areas suggestive of fibroadenomas. Two other discordant phyllodes tumors harbored high epithelium content. The contribution of the epithelial component to the performance of the assay has yet to be ascertained although previous studies have shown that mutations were found in the stromal but not epithelial component [34, 35].

The limitation of our validation cohort is that the sample size for phyllodes tumor is small but the test was validated on a larger number of fibroadenomas, which have higher incidence compared to phyllodes tumors. We incorporated fibroadenomas on core biopsies which were not excised surgically although these may theoretically include uncertainty as the diagnoses are based solely on the core biopsy and not on the excised tumor. However, precluding fibroadenomas without subsequent excisions would result in a selection bias due to the exclusion of a large portion of representative cases. Moreover, the incidence of phyllodes tumor subsequent to a fibroadenoma diagnosis on core biopsy is very low [36], with an average duration of 12 months to the final correct diagnosis.

We do not advocate that the current diagnostic framework be replaced by the assay. Apart from the histological findings, clinical decision whether to proceed with surgical excision takes into account other factors such as radiological size and characteristics, as well as patient symptoms. For instance, a diagnosis of fibroadenoma on core biopsy may still be followed by excision if there is radiologic-pathologic discordance, or if the lesion is large or symptomatic. A diagnosis of phyllodes tumor on core biopsy however, warrants excision. Incorporating the results from our assay allows an additional tool that can be integrated into the decision-making process, enhancing precision especially when it affirms the pathological assessment on core biopsy. The gene assay is also helpful for pathologists in interpreting these lesions when the histological characteristics are indeterminate or ambiguous. This is exemplified by the 22 fibroepithelial lesions without a conclusive classification on core biopsy in the validation cohort. The multigene assay was able to classify 82 % of these cases accurately with a PPV of 94.7 %. The practicality and utility of the assay however, will need to be further validated in prospective studies.

The five-gene assay includes genes of various biological functions. *FN1* (fibronectin 1) encodes a major component of the extracellular matrix. *APOD* (apolipoprotein D) and *ABCA8* (ATP-binding cassette, sub-family A member 8) encode transporter proteins while *PRAME* (preferentially expressed antigen in melanoma) and *CCL19* (chemokine ligand 19) genes are involved in immunoregulatory processes. Some of these genes were reported to be useful in differential diagnosis of other forms of tumors such as *FN1* as a marker for renal cell carcinoma aggressiveness [37], *PRAME* as a marker for differentiating Müllerian carcinoma from malignant mesothelioma [38] and *ABCA8* as part of a multigene gene assay for classifying cancer types [39]. While the individual functional role of these genes has not been implicated in breast fibroepithelial lesions, we found that these markers work best in combination for differential diagnosis between fibroadenomas and phyllodes tumors, as derived from our model algorithm. Nonetheless, it would be of interest to investigate the functional roles of these genes in breast fibroepithelial lesions in future studies.

## Conclusions

We have developed a practical molecular assay for fibroepithelial lesions, classifying fibroadenomas and phyllodes tumors in pre-operative core biopsies. This may serve as an adjunctive aid for accurate pathological diagnosis. Prospective real-world trials will be helpful to determine whether improved surgical decision-making, supported by more accurate histological diagnosis, will lead to better outcomes.

## Additional file

Additional file 1: Supplementary tables. **Table S1** Details of 48 samples from 38 patients constituting the training cohort. **Table S2** Primers designed for potential differentiating genes and normalization genes. **Table S3** Significant genes differentially expressed between fibroadenomas (FAs) and phyllodes tumors (PTs). **Table S4** Correlation between expression profiling and qPCR assays based on a pilot run of six representative samples. Genes with good correlation value (r ≥ 0.6) were subjected to testing on remaining 40 samples. (XLSX 59 kb)

## Abbreviations

*ABCA8*: ATP-binding cassette, sub-family A member 8
AIC: Akaike information criteria
*APOD*: Apolipoprotein D
AUC: area under the curve
*CCL19*: chemokine ligand 19
CI: confidence interval
Ct: threshold cycle
FA: fibroadenoma
FEL: fibroepithelial lesion
FFPE: formalin-fixed, paraffin-embedded
*FN1*: fibronectin 1
H&E: hematoxylin and eosin
HT: high throughput
*NPTX2*: neuronal pentraxin II
NPV: negative predictive value
PPV: positive predictive value
*PRAME*: preferentially expressed antigen in melanoma
pRB: retinoblastoma-associated protein
PT: phyllodes tumor
qPCR: quantitative polymerase chain reaction
RF: Random Forest
*TRIM29*: tripartite motif containing 29
